# A Radiometer for Precision Coherent Radiation Measurements

**DOI:** 10.6028/jres.097.012

**Published:** 1992

**Authors:** Douglas B. Thomas, Edward F. Zalewski

**Affiliations:** National Institute of Standards and Technology, Gaithersburg, MD 20899

**Keywords:** coherent radiation, laser, photocurrent, radiometer, silicon photodiode

## Abstract

A radiometer has been designed for precision colierent radiation measurements and tested for long-term repeatability at wavelengths of 488 and 633 nm. The radiometer consists of a *pn* silicon photodiode maintained in a nitrogen atmosphere with a quartz window designed to eliminate interference problems. Ratio measurements between the radiometer and an absolute type detector were made over a period of 215 d. At 0.5 mW, the standard deviations were 0.008% and 0.009% at 488 and 633 nm, respectively. The maximum deviations from the mean were 0.016% and 0.015% at the respective wavelengths. Measurements were also made on the radiometer with respect to angular and spatial uniformity and linearity. The high precision, simplicity, and portability of the radiometer suggest it for use as a transfer standard for radiometric measurements.

## 1. Introduction

The need for high accuracy radiometric measurements has manifested itself in recent years in military and industrial research and in manufacturing applications. The recent development of high accuracy cryogenic radiometers [1] has made it possible to realize accuracies approaching the 0.01% level. In order to utilize this capability in routine laboratory measurements, a transfer standard is needed with the precision and long-term stability that is better than most portable radiometers currently available for coherent radiation measurements. We have constructed and tested a nitrogen filled, wedged-window radiometer (WWR) for coherent radiation measurements using a Hamamatsu^2^ S1337-1010B *pn* silicon photodiode of the type found to be most stable by Korde and Geist [2]. The superior stability and uniformity of this photodiode is a considerable improvement over previous generation photodiodes. The wedged window is designed to eliminate interference problems associated with detector windows of conventional design. The WWR is a device which can be used in conjunction with an appropriate absolute radiometer for fundamental calibration and does not in itself serve as an absolute device. Radiometers such as the QED-200 discussed below and cryogenically operated electrical substitution devices serve as the fundamental radiometric base with the WWR serving as a useful secondary radiometer for routine usage for some laboratory circumstances. A preliminary version of this paper was reported at an SPIE conference in 1989 [3].

## 2. Description of the Experiment

**Fig. 1:**
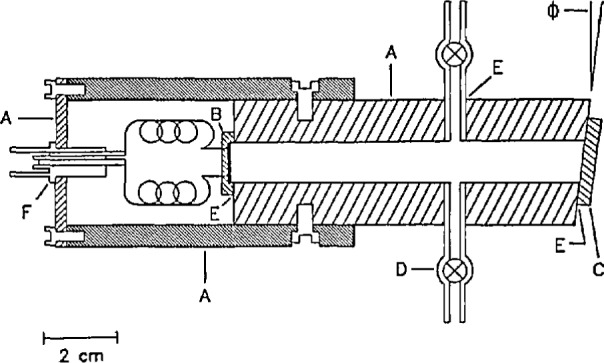
Cross-section of the wedged window radiometer. A, black opaque plastic; B, silicon photodiode; C, quartz wedged window; D, glass stop-cock valve; E, epoxy cement; F, BNC connector; ϕ, 3.8° angle (not to scale).

The basic components of the WWR include a windowless Hamamatsu S1337-1010B *pn* type silicon photodiode, a quartz wedged-window, a heavy wall black plastic tube, and stop-cock valves. The photodiode was specially selected by the manufacturer for spatial uniformity of response and high shunt resistance. Figure 1 is a cross-section drawing of the WWR. The wedged window and the photodiode are sealed at opposite ends of the plastic tube with epoxy cement. The hermetically sealed tube is filled with nitrogen of 99.998% minimum purity (including water vapor) as certified by the manufacturer and then sealed by using two glass stop-cock valves. The nitrogen gas pressure in the tube is slightly above 1.0×10^5^ N/m^2^ (1 atm). The window is 25 mm in diameter with a minimum thickness of 3 mm and a wedge angle of 3.8°. Also, the end of the plastic tube to which the window is sealed is cut at an angle of 3.8° (*ϕ* in Fig. 1). The window is sealed to the end of the plastic tube at an orientation such that the wedged plane of the window is rotated 90° from the 3.8° cut on the plastic tube and thus neither plane of the window is parallel to the surface of the photodiode. This minimizes interference effects.

**Fig. 2:**
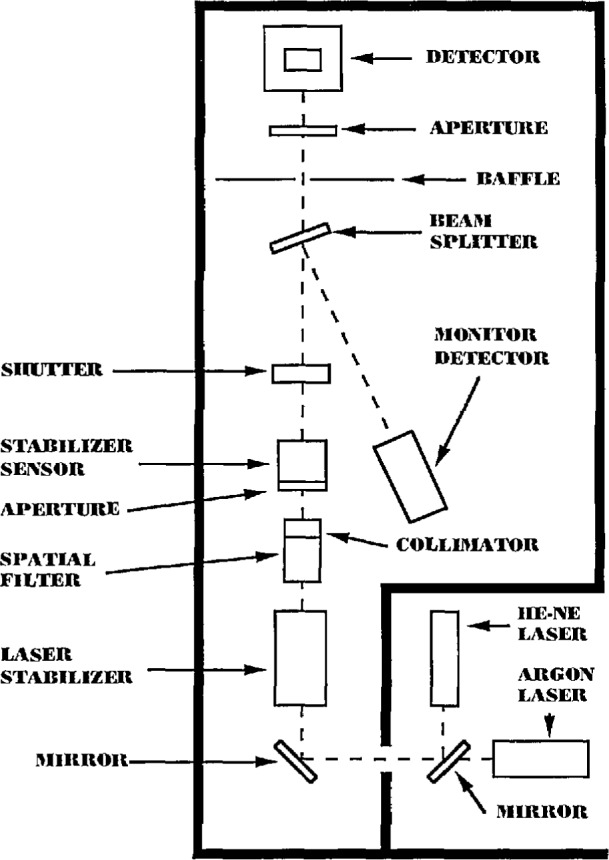
Laser-based detector calibration facility for 483 and 633 nm wavelengths.

Measurements on the wedged-window radiometer were made using the laser-based detector calibration facility shown in Fig. 2. This facility has been carefully constructed to allow for precision measurements on detectors using laser sources. The detectors were tested in a light-tight enclosure and a number of precision positioning devices were used to insure the repeatability of the settings necessary to carry out the high precision measurements discussed here.

The basic components in the facility include two laser sources–a 9 mW helium-neon laser and a 15 mW air-cooled argon laser, a laser power stabilizer, spatial filter, beam expanding telescope, wedged beam-splitter, and a monitor detector. The monitor detector was a Hamamatsu *pn* silicon photodiode similar to the type used in the WWR. Although the monitor diode was not protected by a window, its short-term (<2 h) stability when measured against a QED-200 absolute radiometer (see below) was <0.02%. All measurements of the QED-200 and WWR photocurrents were made as ratios to the monitor detector photocurrent. Thus, the monitor detector needed to be stable only during the short period when the respective photocurrents were measured. If the monitor detector had exhibited significant instabilities during the photocurrent measurements, these instabilities would have been reflected in the final WWR and QED ratios.

The laser power stabilizer was a Cambridge Research and Instrument Company model LS-100, a commercial version of the stabilizer described by Fowler et al. [4]. The internal beam-splitter/sensor of the stabilizer was disconnected and replaced by an external beam-splitter/sensor located after the spatial filter and beam expanding telescope. In this way, the stabilizer controls only that portion of the laser beam selected by the pinhole in the spatial filter. Since the laser beam was vertically polarized, the best stabilizer performance was obtained by mounting the beam-splitter/sensor so that the radiation is reflected horizontally. The variable internal reference voltage of the stabilizer was used to vary laser power over the 0.08 to 0.9 mW range.

The spatial filter was used in a somewhat unconventional way. The filter’s objective lens focused the laser beam slightly in front of the 25 *μ*m pinhole so as to overfill the pinhole. This produces a circular diffraction pattern on the iris diaphragm just before the beam-splitter. The iris diaphragm was adjusted to intercept the first dark ring in the diffraction pattern and a collimator was used to collimate the diffracted laser beam. This eliminated most of the diffraction and scattering effects arising from the iris diaphragm. The final iris diaphragm, in front of the detector comparator position, was adjusted to eliminate any scattered light arising from the first iris diaphragm and the two beam-splitters. The maximum peak-to-peak variation of the laser power over a 30 min period was 0.05%. This was determined using a 100% quantum efficient Model QED-200 absolute radiometer.

An absolute standard generally used in the laser based detector calibration facility is the commercially available 100% quantum efficient detector device [5], Model QED-200, manufactured by the United Detector Technology Company. Ratio measurements between the WWR and a QED-200 were made to determine the long-term stability of the WWR and to determine the linearity of both devices. The acquisition of data was facilitated by the use of a computer programmed to measure the QED or WWR photocurrent followed by a measurement of the monitor detector photocurrent. The ratio of the two photocurrents was then computed. A measurement sequence consisted of 150 repeats of the measurement of the ratio over a time span *of about* 30 *min.* The 150 measurement sequence of the QED to monitor detector ratio was followed immediately by a 150 measurement sequence of the WWR to monitor detector ratio. The ratio of the average of these two measurement sequences represents one data point in the long-term stability discussion in Sec. 4. The integration time for a single photocurrent measurement was 167 ms. All of the long-term stability measurements on the WWR were made (a) at an ambient temperature of 24 ± 0.5 °C, (b) with a laser beam diameter of approximately 4 mm, and (c) with the laser beam normal to and centered on the WWR wedged-window.

## 3. Interference Effects Using Conventional Windows

**Fig. 3:**
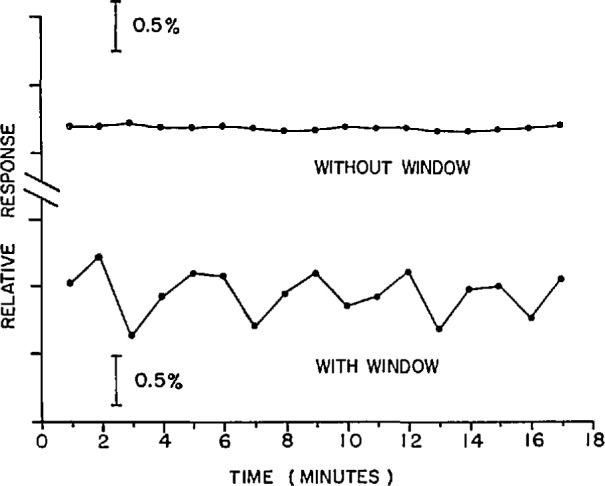
Relative response of a silicon photodiode with and without a window. A 4 mm diameter laser beam at 633 nm and 0.5 mW was positioned normal to and centered on the photodiode surface. The precision of the measurements is 0.016% (1 σ).

The magnitude of interference effects which can occur when photodiodes with conventional windows are irradiated with coherent radiation was demonstrated using the laser-based detector calibration facility. A 0.5° quartz wedged-window approximately 10 mm in diameter and 1 mm thick was mounted on an EG&G UV444B silicon photodiode. The window was mounted such that one surface of the window was parallel to the surface of the photodiode with a separation between the two surfaces of approximately 2 mm. The photodiode (with window) was irradiated with a 4 mm diameter laser beam at 633 nm and 0.5 mW while the photocurrent was measured over a period of 17 min. The laser beam was normal to the photodiode surface. The window was then removed and the photocurrent was measured again. Figure 3 shows the relative response of the photodiode with and without the window. The maximum peak-to-peak change in response with the window was 0.95% and without the window was 0.08%. The response data shown in Fig. 3 raises a question concerning the causes of the larger fluctuations in response with the window in place when compared to the fluctuations without the window. Since measurements were made with both the photodiode and the laser in a fixed position, it would seem that the resulting interference pattern would also be fixed and thus produce a relatively constant photodiode response. However, many He-Ne lasers have beam spatial profiles that change slightly in intensity over short periods of time. Also, these lasers can have spectral mode fluctuations over short periods of time. These phenomena can cause changes in an interference pattern and thus produce response changes in the photodiode.

**Fig. 4:**
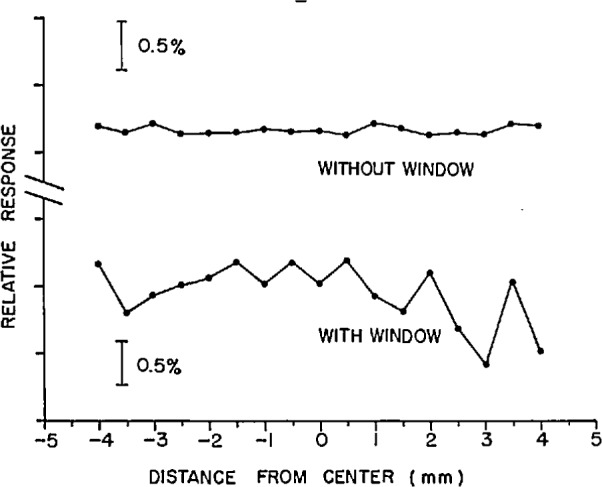
Relative response of a silicon photodiode with and with-out a window. A 4 mm diameter laser beam at 633 nm and 0.5 mW was translated across the diameter of the photodiode with the beam normal to the photodiode surface. The precision of the measurements is 0.016% (1 σ).

It was also found that the window introduced a spatial nonuniformity. Figure 4 shows the spatial variation in response of the photodiode when the laser beam was moved across the diameter of the photodiode in 0.5 mm increments. With the window, the maximum peak-to-peak change in response was 1.21% and without the window the maximum was 0.14%. This clearly unsatisfactory performance is attributed to an interference effect between the window and the reflective surface of the photodiode. If one surface of the window and the surface of the photodiode are approximately parallel, interference fluctuations (resulting in response fluctuations) can occur. In order to eliminate these effects, the wedged-window radiometer (WWR) discussed above and depicted in Fig. 1 was constructed and tested.

## 4. Stability of the Wedged Window Radiometer

**Fig. 5:**
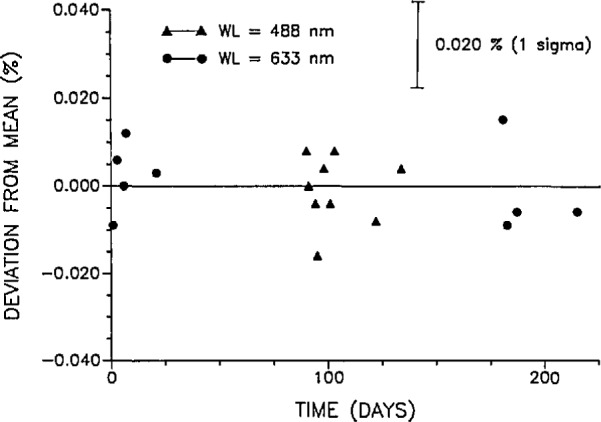
Deviation of all WWR to QED ratios at 0.5 mW from their respective mean at 488 and 633 nm. Each data point represents the average of a 150 measurement sequence. The standard deviations of the ratios are 0.008% and 0.009% at 488 and 633 nm, respectively. The uncertainty in each ratio value is ±0.020% (1 σ) which is the quadrature sum of the standard deviations of the WWR to monitor and QED to monitor ratios.

Measurements of the long-term stability of the WWR were made over a period of 215 d at 633 nm and 40 d at 488 nm. Figure 5 is a plot of the deviations of all WWR to QED measurement-sequence ratios at 0.5 mW from their respective mean at 488 and 633 nm. The maximum deviations from the mean were −0.016% and +0.015% at the two wavelengths, respectively. Each data point in Fig. 5 represents a ratio of the average of 150 measurements of the WWR photocurrent to the average of 150 measurements of the QED photocurrent. The precision in each ratio value presented in Fig. 5 is the quadrature sum of the standard deviations of the WWR to monitor and QED to monitor ratios. This precision is 0.020%. The long-term repeatability of the WWR to QED ratios, i.e., the standard deviation of the ratio values in Fig. 5, is 0.008% at 488 nm and 0.009% at 633 nm. For power levels above and below 0.5 mW, the WWR and QED ratios deviated from the ratio mean at 0.5 mW by a maximum of + 0.115% and +0.155% at 488 and 633 nm respectively. A discussion of these larger deviations is presented in Sec. 6.

## 5. Spatial and Angular Uniformity Measurements

**Fig. 6:**
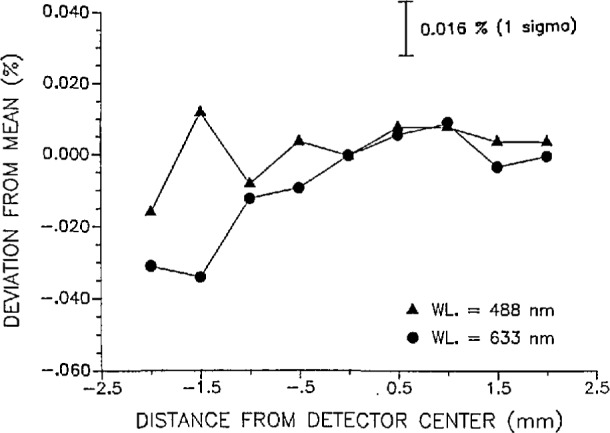
Spatial uniformity of the WWR showing response deviations from the mean response at the photodiode center. The laser beam (4 mm in diameter and 0.5 mW at both 488 and 633 nm) was translated across the diameter of the WWR photodiode with the beam normal to the photodiode surface. The precision of the measurements is 0.016% (1 σ).

**Fig. 7:**
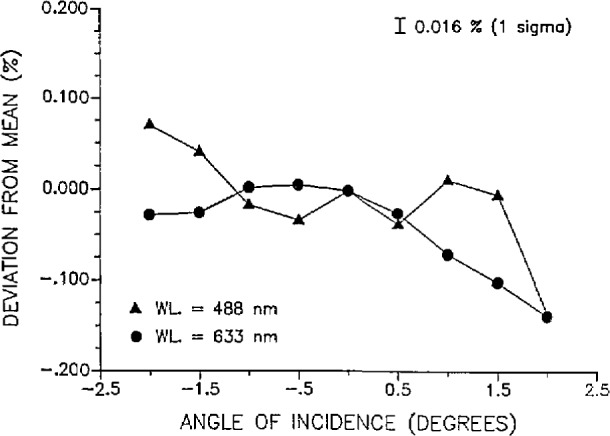
Angular uniformity of the WWR showing response deviations from the mean response when the laser beam is normal to the photodiode. The angle of incidence of the laser beam on the photodiode was changed in 0.5° increments from a beam position normal to and centered on the photodiode surface. The laser beam was 4 mm in diameter and 0.5 mW at both wavelengths. The precision of the measurements is 0.016% (1 σ).

Spatial and angular uniformity measurements were made on the WWR at 0.5 mW at both 488 and 633 nm. The result of the spatial uniformity scan across one diameter of the WWR is plotted in Fig. 6. The maximum deviation of the spatial measurements from the detector center position mean response was 0.034% or about twice the measurement precision of 0.016% (lσ). The angular uniformity measurements are plotted in Fig. 7. The angles listed represent the angular deviations of the laser beam from a line normal to the photodiode surface. Within a 2° range, the maximum deviation from the normal response (response with the beam normal to the diode surface) was 0.139%. Both the spatial and angular uniformity measurements were obtained with a 4 mm diameter beam. Thus, the spatial uniformity of the response of WWR is within a distance of ±1.5 mm from the center of the WWR photodiode. Furthermore, the response of the WWR is constant within 0.060% over an angular displacement of ± 1°.

## 6. Comparison of the QED and WWR at Different Power Levels

From some of our earlier, less precise experiments with the QED-200, we had an indication that these devices might be nonlinear under reverse voltage bias at power levels above 0.5 mW. In conversations with L. P. Boivin of the National Research Council of Canada and with C. R. Duda of the United Detector Technology Company, we learned that they also observed what appeared to be small nonlinearities under similar measurement conditions. Since the QED-200 is now being applied in many high accuracy radiometric measurements, we have decided to report our limited observations in order to encourage further studies. Kohler, Pello, and Bonhoure report similar nonlinearity for the QED in this study of temperature effects on the device [6].

**Fig. 8:**
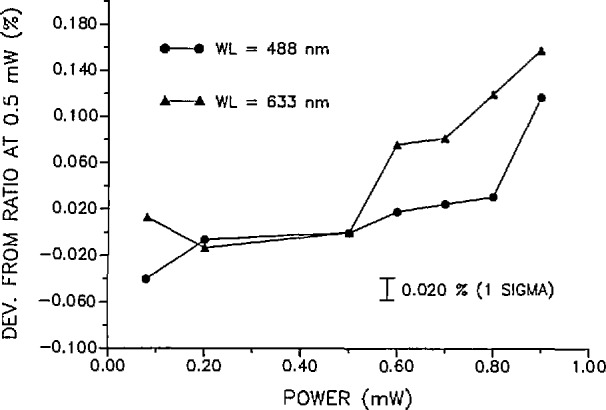
Deviations of the QED/WWR ratios at various radiant power levels from the QEDAVWR ratio at 0.5 mW at both 488 and 633 nm. The uncertainty in each ratio value is ±0.020% (1 σ) which is the quadrature sum of the standard deviations of the WWR to monitor and QED to monitor ratios.

Measurements were made of QED/monitor ratios and WWR/monitor ratios at various radiant power levels from 0.08 to 0.9 mW. The WWR/monitor ratios over this power range were constant (within the precision of the measurements) while the QED/monitor ratios showed a general increase above 0.5 mW. Figure 8 shows the deviation of the QED/WWR ratios at various radiant power levels from the QED/WWR ratio at 0.5 mW. The figure shows the ratios to be generally increasing at both wavelengths with increasing power. However, between 0.08 and 0.5 mW at 633 nm and between 0.2 and 0.5 mW at 488 nm, the ratios appear to be constant within the limits of the measurement uncertainty. No measurements were made above 0.9 mW since this was the maximum power attainable with the system. It should be noted that the measurements were made with reverse bias voltages on the QED of 30 and 10 V at 633 and 488 mn, respectively.

Although the QED/monitor and WWR/monitor measurements indicated the QED to be nonlinear, we felt it necessary to conduct an experiment to show that the nonlinearity could not be attributed to polarization changes in the laser beam at the various power levels.

During the course of the described experiments, the laser power was varied by changing the voltage applied to a birefringent crystal in the laser stabilizer. This causes a change in the polarization of the laser beam and a subsequent change in the amount of power transmitted by the polarizer located in the exit port of the laser stabilizer. If this polarizer was not perfect, the resulting beam polarization may not be 100% linearity polarized as expected. The QED consists of three detectors, two of which are oriented at a 45° angle to the laser beam. The WWR has a window with both surfaces oriented slightly off normal to the laser beam. The differences in the polarization sensitivity of these two devices might account for the differences in their response.

To check the degree of polarization of the laser beam exiting the stabilizer, a prism polarizer was placed in the beam and its vertical-to-horizontal transmission was measured at 633 nm. The detector chosen for the polarization variation measurement was a Hamamatsu S1227-1010B without a window. The uniformity of this detector was measured and found to vary less than 0.1% over the central 6 mm diameter area. It was aligned normal to the laser beam and checked for possible polarization sensitivity by rotating it around the laser beam axis. The variations were within the 0.016% precision of the measurement.

At 633 nm the vertical-to-horizontal polarization ratios of the laser beam were found to change slightly at different power levels. At 0.08 mW, the ratio was 7,300 to 1; at 0.5 mW, 9,400 to 1; and at 0.9 mW, 10,800 to 1. Thus, there is a change in the polarization of the laser beam, but the horizontal component is never greater than 0.014% of the vertical component of the beam. Therefore, the change in the polarization of the beam cannot account for the power dependent differences between the QED and the WWR.

The Hamamatsu S1227-1010B detector was also used in comparisons with the WWR and the QED at 633 nm and power levels of 0.08 mW, 0.5 mW, and 0.9 mW. The ratios of the WWR to the Hamamatsu detector were constant to within the precision of the measurements for the three power levels. For the QED versus the Hamamatsu detector measurements, an increase of 0.133% was observed for the QED between the 0.5 and 0.9 mW power levels. This value agrees quite well with the increase of 0.155% for the QED versus the WWR as indicated in Fig. 8 at the same power levels.

Since small ambient temperature changes can affect the spectral response of photodiodes, this phenomenon was considered as a possible source of instability in the three types of diodes used in this study. The maximum ambient temperature fluctuations in the measurement facility during one measurement cycle (1 h) was ±0.5°C. The ambient temperature of the laboratory was 24 °C. Using temperature coefficient data we measured on Hamamatsu S1226-1010B and S1337-1010B photodiodes, the response change in these diodes at 488 and 633 nm is less than 0.01%/°C. Information received from the manufacturer of the QED-200 radiometer lists a temperature coefficient of less than 0.1%/°C for the diodes in this radiometer. Thus, the effects of temperature on the diodes used in this study are too small to explain the observed nonlinearity of the QED-200 radiometer.

## 7. Conclusion

Ratio measurements between the WWR and the QED over a period of 40 days at 488 nm show the repeatability (1σ) of the differences between the ratios and their mean to be 0.008%. At 633 nm over a period of 215 d, the repeatability (lσa) of the ratio differences was 0.009%. This repeatability was determined at 0.5 mW. The maximum deviations of the ratios from the mean of all ratios at the respective wavelengths were −0.016% and + 0.015% at 488 and 633 nm respectively. At power levels between 0.08 and 0.9 mW, the response of the WWR appears to be linear with power. However, the QED appears to exhibit some nonlinear behavior at power levels above 0.5 mW.

Measurements on the spatial and angular uniformity of the WWR at 488 and 633 nm showed that the WWR is uniform to within the ±0.016% measurement precision for translations within ±1.5 mm from the detector center. This indicates that with a careful selection of photodiodes, good uniformity can be obtained for precision measurement purposes. Also, the WWR exhibited angular uniformity within ± 0.060% for angle deviations from the normal up to ±1°. These measurements indicate that the highest precision is achieved with the WWR when it is calibrated and used with (a) a 6 mm diameter or smaller laser beam, (b) the beam located in the same position on the photodiode surface, and (c) the beam optical axis normal to the photodiode surface. This implies that care must be taken in performing measurements with this device to achieve the precision reported here. This care not only requires selection of the photodiode but the maintenance of a precision optical measurement facility.

Since the WWR is not an absolute radiometer, it must be calibrated using an absolute standard. Such standards include (a) the 100% quantum efficient detector radiometer (QED), (b) a self-calibrated silicon photodiode [6] [8], (c) a cryogenic absolute radiometer [9], (d) a cavity-type electrically calibrated radiometer [10] and (e) an electrically calibrated pyroelectric radiometer (ECPR) [11]. The reported absolute accuracies of these standards range from 0.01% for the cryogenic radiometer to 0.7% for the ECPR.

It should be emphasized that the repeatability of the WWR reported in this paper represents the performance of one specific device in a measurement facility where variables were carefully controlled. If another sample of the device were calibrated and used in a facility where parameters such as temperature, beam size, and beam uniformity vary, the repeatability of measurements made with the device could be significantly different.

The manufacturer of the photodiode used in the WWR reports a “worst-case” temperature coefficient for the diode of 0.2 %/°C as compared to the very small temperature coefficient measured for the device used in the present measurements.

If the size of a beam used to irradiate the WWR varies from one measurement to another, this would affect the measurement repeatability. Using the uniformity values reported in Fig. 6, it can be seen that a beam diameter change from 1.0 to 3.0 mm could result in a “worst-case” response change of 0.035% at 633 nm.

The effects of beam uniformity on the repeatability of the WWR would be similar to the effects discussed for beam size. For example, if the spectral response of the WWR were determined with a laser beam which had a “hot-spot” (a portion of the beam with higher intensity than the average intensity of the beam) in the first quadrant of the beam cross-section and again determined with a “hot-spot” in the third quadrant, the two response values could be different due to diode non-uniformity. If the uniformity values reported in Fig. 6 can be considered as typical, the “worst-case” effect due to beam nonuniformity would be a response change of 0.035% at 633 nm.

Although it is unlikely that all of the above “worst-case” situations would occur during a specific WWR measurement sequence, a quadrature summation of these effects reveals a total possible error of 0.21%, assuming a one degree variation in temperature. Thus, the above analysis shows that special care must be taken to control measurement variables and the temperature coefficient of the specific device must be measured in order to realize the measurement precision capabilities of the WWR.

If the WWR were calibrated to the accuracy attainable using the cryogenic absolute radiometer and if measurement variables are carefully controlled, it is not unreasonable to expect the calibration to have an absolute uncertainty of 0.02% to 0.03%. If this is achievable, the WWR would provide a low-cost, portable and stable radiometer for a variety of radiometric purposes, particularly in circumstance where the ambient air was subject to humidity and temperature variation.

## Footnotes

2 Certain commercial equipment, instruments, or materials are identified in this paper to specify adequately the experimental procedure. Such identification does not imply recommendation or endorsement by the National Institute of Standards and Technology, nor does it imply that the materials or equipment identified are necessarily the best available for the purpose.
